# Effectiveness of a Brief Functional Analysis and Functional Communication Training Conducted Through Telehealth

**DOI:** 10.1007/s10882-022-09857-6

**Published:** 2022-08-08

**Authors:** Emma A. Craig, Katerina Dounavi, Jenny Ferguson

**Affiliations:** School of Social Sciences, Education & Social Work, Queen’s University of Belfast, 69-71 University Street, Belfast, BT7 1HL Northern Ireland

**Keywords:** Telehealth, Brief functional analysis, Functional communication training, Challenging behaviour

## Abstract

This study evaluates the effectiveness of a brief functional analysis and functional communication training conducted via telehealth. Three interventionist-child dyads took part in the study including one speech and language pathologist and two school teaching assistants, each working with one child with autism spectrum disorder. Interventionists were trained using didactic training to implement a brief functional analysis as well as synchronous coaching from a BCBA^®^ to implement functional communication training. A multiple baseline across participants design was utilised to evaluate if interventionists could implement functional communication training to decrease challenging behaviours that included aggression, elopement and disruption. Sessions concluded earlier than planned due to school closures mandated by the COVID-19 outbreak for two of the three participants; however, existing data provide evidence that telehealth is a valid model for enabling clinicians to work in collaboration with school personnel to effectively deliver assessment and intervention procedures remotely via telehealth.

Many children with autism spectrum disorder (ASD) engage in challenging behaviours such as self-injury, aggression, and disruption (Dominick et al., 2007). Challenging behaviour in children with ASD, if left untreated, can lead to a poor quality of life, isolation from social relationships and exclusion from typical home environments, supported employment and educational settings (Department for Education, 2019). Following a vicious circle, exclusion from meaningful life activities often leaves behaviour open to worsen rather than improve (Baker et al., 2005; Horner et al., 2002; Morano et al., 2017). Further efforts need to be made to ensure that children with ASD across the globe who engage in challenging behaviours are receiving evidence-based interventions aimed at eliminating challenging behaviours while increasing socially valid skills.

Functional analysis (FA) is an experimental method used to determine which environmental factors maintain challenging behaviour (Iwata et al., 1982, 1994). Once a function for the challenging behaviour is identified, practitioners can design function-based interventions to decrease challenging behaviours (Dounavi, 2011). Intervention packages that have been designed based on individualised FA results often consist of differential reinforcement of alternative behaviours that match the function of challenging behaviour, such as functional communication training (FCT); such packages have consistently shown positive outcomes at reducing or eliminating challenging behaviours (Carr & Durand, 1985; Fisher et al., 1993; Tiger et al., 2008).

It is expected that the person designing and supervising assessment and intervention procedures for individuals with ASD is qualified (e.g., as a Board Certified Behaviour Analyst or BCBA^®^). Although there is an increase in the number of BCBAs^®^, this appears to be more apparent to those residing in the United States (US) (Tsami et al., 2019). In countries outside of the US, particulary within Europe and beyond, access to such professionals is extremely limited (Craig et al., 2021; Dounavi et al., 2019). This reality has made access to quality behaviour analytic services limited to a few individuals and their families (Keenan et al., 2015). To add to the difficulty in locating a BCBA^®^ with availability in countries outside of the US, the recent change in the accessibility of certification to non-US residents introduced by the Behaviour Analyst Certification Board (BACB^®^) (BACB, 2019) has posed further obstacles for individuals to receive training, supervision or intervention from an appropriately qualified professional. With the limited number of appropriately qualified professionals, training caregivers and entry-level professionals to high fidelity has the potential to bring interventions to a much wider community of individuals diagnosed with ASD and their families across the world. Such training in evidence-based assessment and intervention procedures that have shown to work face-to-face (e.g., Tanner & Dounavi, 2020) can also prevent interventionist burnout through ongoing support (Dounavi et al., 2019). Therefore, research examining the implementation of assessment and intervention procedures using alternative service delivery platforms, such as telehealth, is needed in an international context.

Telehealth is the use of communication technology, such as video-conferencing, for the delivery of health-related services (World Health Organization, 2022). Telehealth enables patients and clinicians to receive services from a distance in a cost and time efficient manner and making evidence-based practice more widely accessible. Telehealth has already shown success with patients suffering from dementia to heart disease and diabetes (Kessler et al., 2009; NHS, 2022; Webb et al., 2010). Since the outbreak of COVID-19, individuals have been unable to leave their homes resulting in fundamental behaviour analytic services having to cease. With the virus affecting 213 countries (World Health Organization & International Telecommunication Union, 2022), practitioners have been seeking alternative delivery methods such as telehealth to continue offering services to populations in need. The pandemic has highlighted the need for further research on the effectiveness and acceptability of telehealth as a way to enable practitioners to continue with the delivery of assessment and intervention procedures efficiently.

To date, the literature surrounding telehealth has shown positive outcomes (Craig et al., 2021; Ferguson et al., 2019, 2022a, 2022b). Since the outbreak of COVID-19, numerous studies have evaluated the use of telehealth to provide behaviour analytic interventions to teach a number of skills to individuals with ASD such as functional living skills (Boutain et al., 2020; Craig et al., 2021; Gerow et al., 2021a, 2021b), communication, (D’Agostino et al., 2020; Ferguson et al., 2022a, 2022b), and tolerating wearing a face mask (Sivaraman et al., 2021). Further, research before and during COVID-19 has also examined telehealth as a platform that facilitates functional behaviour assessments such as FA (Schieltz & Wacker, 2020). An initial examination of the literature shows postive results when conducting traditional FA via telehealth and studies have shown a reduction in challenging behaviour with FCT based on FA results (Lindgren et al., 2016, 2020; O’Brien et al., 2021a, b; Tsami et al., 2019) with gains generalising beyond the training setting (O’Brien et al., 2021a, b). Tsami et al. (2019) examined the effectiveness of parent training via telehealth among families around the world. Results suggested that training parents in assessment and intervention procedures such as FA and FCT was effective for reducing challenging behaviour. A recent literature review found that the evidence for behaviour assessments conducted via telehealth is sufficient (Neely et al., 2021); however, the assessment procedures examined have typically focused on traditional FA methodologies as opposed to procedures such as brief FAs, which have limited evidence of efficacy when conducted through telehealth.

In a brief FA, each experimental condition is presented for a shorter period of time allowing researchers to identify behaviour function in a more efficient manner (Northup et al., 1991). The brief FA methodology is well suited to environements where assessment time is limited, such as schools or homes. In a noticeable study, researchers trained parents to implement a brief FA via telehealth with promising results (Gerow et al., 2021a, b). Gerow et al. (2021a, b) found that for four participants, a brief FA was effective at identifying behaviour function. However, a full FA was needed with a fifth participant. The brief FA identified a false function for one participant which subsequently resulted in further assessment procedures. The literature surrounding brief FAs conducted via telehealth indicates that false positive or false negative results are not uncommon (Suess et al., 2016; Wacker et al., 2004), therefore further research is required to refine procedures. Additionally, conducting FA via telehealth where assessment time is often limited, is a topic that should be investigated further.

Few research studies have examined training professionals working with individuals with ASD in brief FA procedures via telehealth, as the majority of previous studies have focused on training parents (Gerow et al., 2021a, b; Suess et al., 2016). Further research examing the effectiveness of conducting brief FAs via telehealth is needed, especially when such procedures can be effective within a limited time period (Gerow et al., 2021a, b). Suess et al. (2016) were able to identify behavioural function through the use of a brief FA in just one appointment, indicating that the brief FA may be suited to telehealth consultations. Furthermore, research examining brief FAs comes primarily from the US where access to qualified professionals is easier compared to Europe and beyond (Craig et al., 2021). Therefore, research that examines assessment and intervention procedures using a telehealth model on an international level is a much needed addition to the current literature.

As such, our study aimed to add to the current literature on an international context by investigating the use of brief FAs conducted via telehealth by professionals working with children with ASD. Specifically, the aims of the study were to assess: (1) if non-US based interventionists can run a brief FA while coached live solely via telehealth, (2) if conducting a brief FA soley via telehealth is effective at determining the variables maintaining challenging behaviour, (3) if interventionists can implement FCT with high fidelity after receiving training solely via telehealth, and (4) if challenging behaviours can be reduced through FCT overseen via telehealth by a BCBA^®^ residing outside of the US.

## Method

### Participants and Setting

Three interventionist-child dyads took part, with two of the interventionists having been involved in a previous study (Jana and Majidah) (Craig, et al., 2021) and a third interventionist, Fadimah, being new to our telehealth lab but working in the same organisation as Majidah. Fadimah and Majidah were teaching assistants working for a school in the United Arab Emirates; they each worked one-to-one with a child with ASD. Jana was a speech and language pathologist working for a clinic in Serbia; she also ran one-to-one sessions with a child with ASD (see Table 1 for demographics). Interventionists and their respective child-participants were from the same culture with the exception of Majidah and Daniel. Daniel was a British Citizen living in the UAE and Majidah was originally from the UAE.

**Table 1:** Participants demographics

Interventionist-child dyads	Age	Gender	Diagnosis	Country and Language	Education	Job Title	Years working with children with ASD
Fadimah	36 years	Female		From the UAE, fluent in English	Bachelor	Teaching Assistant	2 years
Sasmita	5 years	Female	ASD				
Majidah	26 years	Female		From the UAE, fluent in the English	SEN* Diploma	Teaching Assistant	1 year 8 months
Daniel	4 years	Male	ASD				
Jana	33 years	Female		From Serbia, fluent in English	Master	Speech and Language Pathologist	7 years
Catherina	4 years	Female	ASD				

SEN: Special Educational Needs

Interventionists were eligible for the study if: (1) they worked with a child with ASD, (2) were not related to the child other than through a professional relationship, and (3) had no prior formal training in behaviour analysis with the exception of the training on the principles of ABA offered through our telehealth lab in the previous study (Craig, et al., 2021). Children were eligible for the study if they: (1) had a formal diagnosis of ASD as assessed through formal tools such as the Autism Diagnostic Observation Schedule (ADOS-2; Lord et al., 2012), (b) were between the ages of 24 and 96 months (2–8 years), and (c) displayed challenging behaviour such as aggression, self-injury, or classroom disruption.

Sessions took place in the classroom (Majidah and Fadimah) or the room where regular speech and language therapy sessions took place (Jana). The BCBA^®^ (first author) conducted telehealth sessions in a secure office located in Belfast, Northern Ireland (approximately 4,890 miles away from the UAE and approximately 1,860 miles away from Serbia). She had 10 years of experience in the field of ABA and ASD and conducted this work within her doctoral studies focusing on the effectiveness and acceptability of telehealth. The study was supervised by a BCBA-D^®^ and doctoral-level supervisor with nearly 20 years of experience in ABA and ASD and over 10 years of experience designing and delivering telehealth services across numerous countries (second author).

### Equipment and Materials

Sessions were conducted using a MacBook Pro with a built-in microphone and camera. Participants used either a laptop or a smartphone. Participants who did not possess the necessary equipment were sent a Logitech C920 HD Pro webcam and ear buds (JBL T110BT In-Ear Wireless Headphones). Four didactic training PDF documents were designed using Canva (an online design software). SurveyMonkey^®^ was used for weekly quizzes to assess content mastery within the didactic training component. A GoPro™ camera was used to record skills being modelled in the training documents.

Videoconferencing software included Zoom and Skype™, while QuickTime Player was utilised for screen recording purposes. Dropbox™ and WeTransfer were employed for sending and receiving video files. All software utilised was available free of charge, with the exception of Dropbox™ (a free software for interventionists that required a monthly subscription from the BCBA^®^ to obtain a larger storage space), and used encryption. Contents were accessible only through a direct link. Once transferred to the research team, data were stored on a password-protected hard drive which was subsequently backed up onto the University’s secure network.

### Design

A multi-element design was used to evaluate the results of the brief FA. A multiple probe across participants design was used to evaluate the effectiveness of FCT at reducing challenging behaviour. The study was conducted in three phases: (1) baseline of target appropriate behaviour, (2) brief FA of challenging behaviour, and (3) functional communication training. In order for interventionists to meet the mastery criterion, they were required to score 80% or higher on the treatment fidelity checklist over 3 consecutive days. Mastery of child behaviours was set at zero occurrences of challenging behaviours over 3 consecutive days.

### Interobserver Agreement (IOA)

The first and third authors calculated IOA data on 66% of FA and 33% of baseline and FCT sessions across both interventionist and child behaviours. IOA for the occurrence of challenging behaviour was calculated using a mean count per interval IOA formula. IOA was calculated across all FA conditions and participants and averaged 96% (range, 91% to 99%). IOA on the occurrence of challenging behaviour and the number of independent FCT responses (mands) was calculated on 33% of baseline and treatment sessions using a total count formula (Cooper et al., 2007) during the FCT through live training phase. Challenging behaviour IOA averaged 99% (range, 98% to 100%) and total independent mands IOA averaged 99% (range, 98% to 100%). IOA was calculated on the number of FCT steps scored correctly and incorrectly on the fidelity checklist by interventionists using a total count procedure (Cooper et al., 2007) across each interventionist. IOA scores averaged 97% (range, 88% to 100%).

### Dependent Variables

The first author completed a Functional Assessment Screening Tool (FAST; Iwata et al., 2013) with the interventionists before the brief FA which led to the formulation of hypotheses for the variables maintaining challenging behaviour. Challenging behaviours included aggression (Daniel), elopement (Catherina) and disruption (Sasmita). Aggression was defined as any instance of hitting, kicking, biting or pinching. Elopement was defined as moving or attempting to move more than 5 feet away from the teacher or leaving the session room. Disruption was defined as crying or screaming with or without tears and flopping to the floor. Frequency was used to measure child challenging behaviours during the brief FA and FCT. Interventionist treatment fidelity data were not collected during the brief FA as the FA was coached live by a BCBA^®^, therefore interventionist behaviour would have been influenced by the guidance of the BCBA^®^. Additionally, for safety, interventionists would typically not implement an FA without the presence of a BCBA^®^ who is skilled in conducting FA.

During the FCT phase, treatment fidelity was measured during baseline and live coaching sessions against a pre-designed treatment fidelity checklist (Table 2). Interventionists either scored correct or incorrect in each step and the percentage of steps performed correctly was calculated by dividing the total number of correct steps by the total number of steps. Interventionists were required to meet a mastery criterion of 80% or higher on the treatment fidelity checklist over three consecutive days. Challenging behaviours measured in the brief FA were measured during the FCT phase using frequency. Mastery criteria for child behaviours were set at zero occurrences of challenging behaviour over three consecutive days. Independent mands were defined as any instance of requesting a reinforcer appropriately without the need for verbal or physical prompts, also measured using frequency.

**Table 2:** Treatment fidelity checklist

	Sets up environment appropriately (materials such as ‘my way’ card available, and tasks available) Creates appropriate motivating operation (putting child in a state of satiation for an aversive task and/or deprivation for attention) Provides immediate reinforcement for appropriate mand Does not provide demands during child’s free time (and/or provides enriched attention during free time) Prompts FCT response when required Places challenging behaviour on extinction
	Percentage of correct steps:

### Procedure

Due to Majidah and Jana’s involvement in our previous study (Craig, et al., 2021), they had already received training in the following topics: (1) an introduction to applied behaviour analysis, (b) the ABCs of behaviour analysis, (3) reinforcement, (4), extinction (5), motivating operations and verbal behaviour, (6) task analysis and chaining, (7) prompts and prompt fading, (8) data collection and, (9) generalisation. The purpose of this training was to learn the principles of behaviour analysis in order to teach functional living skills to children with ASD. As Fadimah was not involved in the previous study, she had not taken part in the above training topics. Therefore, before beginning the study, Fadimah received the same training on these topics from our previous study as the other two interventionists.

#### Baseline

Baseline data were collected prior to interventionists receiving didactic training to ensure the didactic training did not influence interventionist behaviour. The interventionists recorded three or four 10-min videos from three to four different days showing the child engaging in challenging behaviour and were instructed to respond to the behaviour as they normally would. Interventionists were instructed to record baseline sessions during times of the day when challenging behaviours likely occurred (e.g., during task instruction/removal of preferred items after break time). Interventionists were not instructed to create situations to evoke challenging behaviour during baseline but rather record footage during times of the day where challenging behaviour was most likely to occur. Reasons for using videos recorded in the natural environment for baseline data collection instead of FA data was to ensure that interventionist behaviour was not altered in response to challenging behaviour which would have been the case if baseline data were collected during the FA with synchronous coaching from a BCBA^®^. Using video footage recorded prior to any training or intervention component being in place allowed researchers to avail of a true representation of child challenging behaviour and of how interventionists responded in the natural environment. Data during baseline videos were recorded on the frequency of challenging behaviours, while interventionists’ behaviour was scored on the treatment fidelity checklist.

#### Didactic Training

Interventionists completed a total of four didactic training sessions with the BCBA^®^ (first author), with each session lasting approximately 60 min. The four didactic training sessions were not part of our previous study and were created for the purpose of the current study. All interventionists participated in the didactic training. Two days before each session, interventionists were sent a PDF document designed specifically for this study and instructed to read through the material. Once complete, the interventionists met with the BCBA^®^ using their preferred method of videoconferencing software (i.e., Zoom or Skype™) for a duration of 60 min. Interventionists took two didactic sessions per week. Week one consisted of didactic training covering functional behaviour assessments during the first session and preference assessments during the second session. Week two included one session covering FA and a second session covering FCT.

Upon the interventionist and BCBA^®^ meeting via videoconferencing, the BCBA^®^ worked through the document with the interventionist, with the BCBA^®^ using video modelling from existing videos freely available online and role play to model appropriate skills and answer any questions. For example, during the preference assessment session, the BCBA^®^ read through the training material with the interventionist which provided a description on how to conduct a preference assessment, presented a video model of footage showing a preference assessment being conducted and finally, the BCBA^®^ and interventionist role played a preference assessment procedure. Following the video conferencing session, the interventionists took a quiz to assess content mastery based on the content taught in the didactic training session. Interventionists were required to score 80% on the quiz before moving on to the next topic. If mastery was not met, extra videoconferencing sessions were scheduled until a mastery score of 80% on the quiz was achieved. This process was repeated for all following four didactic training topics.

#### Brief FA

Interventionists met with the BCBA^®^ and BCBA-D^®^ (first and second authors) via videoconferencing to conduct the FA. The brief FA was conducted during one session via telehealth where the BCBA^®^ and BCBA-D^®^ coached interventionists live through the procedures, providing immediate feedback. Each condition lasted 5 min. However, for Daniel, 5-min conditions showed behaviour occurring at a low frequency. A second FA was conducted with Daniel in which condition length was extended to 10 min, capturing a higher frequency of challenging behaviour. During the escape condition, interventionists were instructed to provide difficult and non-preferred demands to the child. On the occurrence of challenging behaviour, interventionists responded with a statement such as “okay, we do not have to do it” and removed all work materials. The child was then given a 30-s break. If challenging behaviour continued to occur during the reinforcement period, demands were not represented until challenging behaviour had ceased for at least 30-s. After 30-s had passed without any challenging behaviour, demands were represented. During the attention condition, the teacher presented the child with a statement such as “I have some work to do” and did not provide the child with any attention. Contingent on challenging behaviour, the interventionist provided a light verbal reprimand, such as “Do not do this”. During the 30-s reinforcement period, attention was freely available. After 30-s had passed without any challenging behaviour, the interventionist returned to her work and refrained from offering any more attention unless challenging behaviour was shown again.

The alone condition was not implemented for Sasmita and Daniel as results from the FAST suggested a social function of challenging behaviour. During the alone condition for Catherina, the teacher left the room but kept the camera on for the first and second author to observe if the challenging behaviour occurred. The interventionist kept her wireless Bluetooth headphones on so that the research team could inform her should she need to return to the room for safety reasons. During the play condition, the children were given free access to preferred items and attention was provided on average every 30-s in the form of verbal praise such as “I love how nicely you are playing”. Due to the sensitive nature of assessments evoking challenging behaviours, interventionist treatment fidelity was not measured during the brief FA as they were coached through the entire process by the BCBA^®^ and BCBA-D^®^.

#### FCT through Synchronous Coaching

Baseline for the FCT phase was collected from baseline video footage sent to the BCBA^®^ prior to didactic training. Interventionists met the BCBA^®^ once per week for a duration of 1-h each time. The first 10–15 min of the session was spent discussing the procedures or answering any questions the interventionist had. The child was available for 45-min during the session where the interventionist was trained to implement FCT procedures. Sasmita and Catherina were taught the vocal verbal response “break” that led to escape from demands. Daniel was taught the verbal response “my way” using a ‘my way’ card which led to escaping from demands and gaining attention. Sessions began with the child playing with preferred items. Interventionists then prompted returning to work (e.g., “It’s time to work.”) and provided an echoic or hand-over-hand prompt for the child to request for a break/my way. Once an appropriate mand was emitted, the interventionist provided reinforcement in the form of escape from demands, (i.e., “sure, you can have a break” for Sasmita and Catherina and frequent attention in the form of verbal praise and escape from demands for 1–2 min for Daniel). The children were not required to complete any tasks prior to receiving access to the reinforcement. Mands were reinforced upon each occurrence. Therefore, when prompted to return back to the task after the break period had ended, if the child manded “break” or “my way” reinforcement was immediately provided. If challenging behaviour occurred during the FCT condition, reinforcement was not provided and a hand-over-hand prompt or a partial physical prompt from the elbow was used to guide the child to complete the task. As the child learned the contingency, prompts for mands were faded until the child manded for the reinforcer independently.

## Results

### FA

**Fig. 1:**
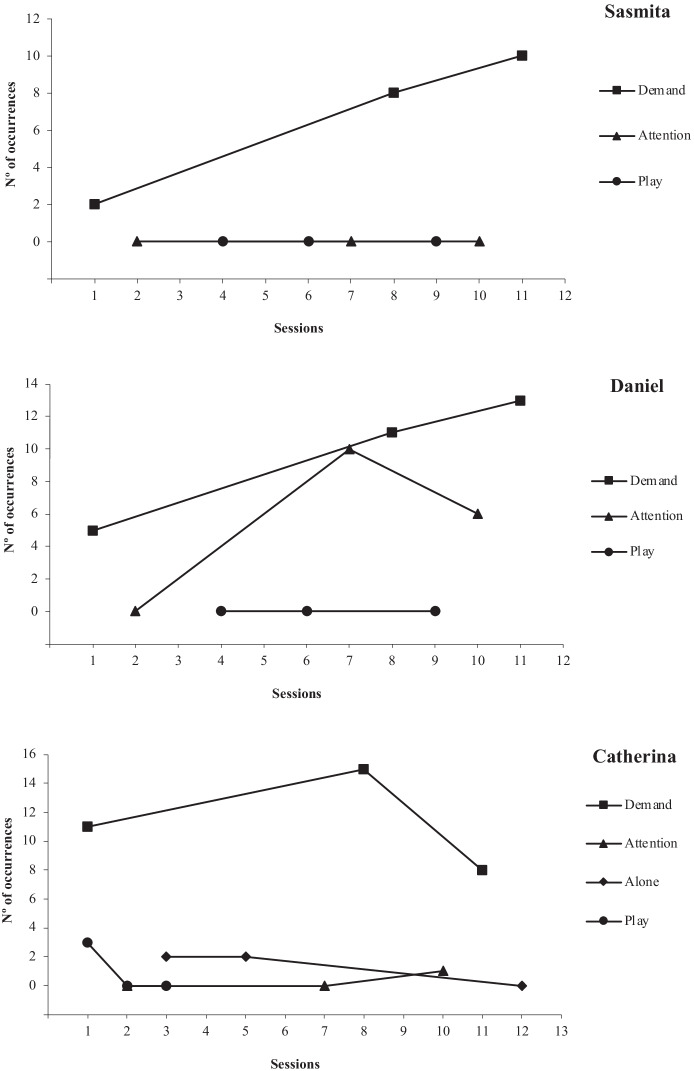
FA Results

All three FAs demonstrated clear maintaining variables for challenging behaviour (Fig. 1). Sasmita’s results illustrated a function of escape showing an increasing trend of challenging behaviour across three demand sessions with an average of six behaviours per session. Challenging behaviour did not occur during the attention and play conditions. Daniel’s FA results also showed an increasing trend during the demand condition with an average of 10 occurrences of challenging behaviour per session. Daniel also engaged in the challenging behaviour during the attention condition, showing an average of five occurrences per session, although the overall level of responding was lower and more instable during the attention condition compared to the demand condition. Daniel’s results suggested a multiple function of challenging behaviour serving both to escape from tasks and gain attention. Catherina’s results showed a clear escape function with challenging behaviour occurring an average of 11 times per session. A few occurrences of challenging behaviour were noted in other conditions, however, these were shown at a low frequency and then decreased to zero, suggesting that once discrimination between conditions was established, challenging behaviour ceased under non-relevant environmental conditions.

### FCT

**Fig. 2:**
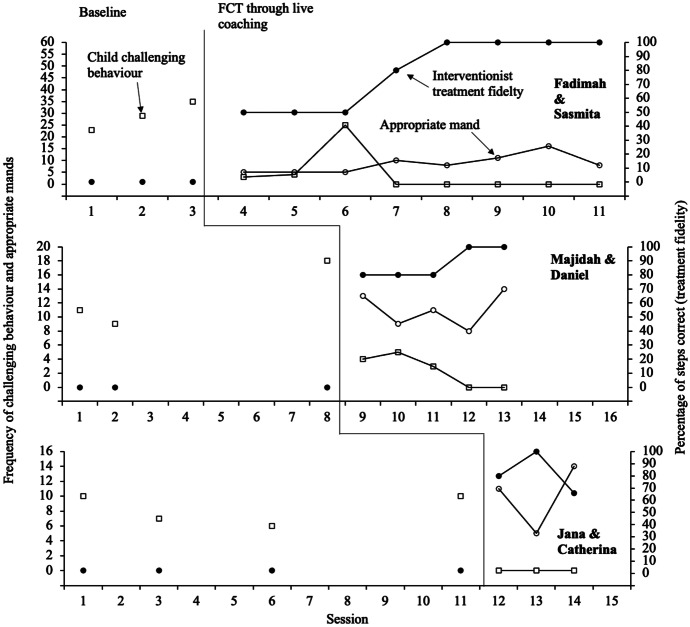
FCT Delivered through Live Coaching

Figure 2 illustrates child results during FCT, including independent mands and challenging behaviour. Sasmita’s baseline levels showed an increasing trend of challenging behaviour (*M* = 29 responses, range, 23 to 35), as did Daniel’s (*M* = 13 responses, range, 9 to 18). Catherina’s baseline measurements illustrated stable responding (*M* = 8 responses, range, 6 to 10). A dramatic decrease in challenging behaviour was shown during the first session of live coaching for all three participants. In contrast, a dramatic increase was illustrated in Sasmita’s challenging behaviours after session three, occurring at a frequency of 25. This was the result of Fadimah scoring 50% in the treatment fidelity checklist in this session, incorrectly restricting access to preferred items during the reinforcement period. By session four and for the remainder of sessions, Sasmita and Daniel’s challenging behaviours occurred at a frequency of zero.

Overall, Sasmita independently manded for a break on an average of 10 times across all live coaching sessions (range, 5 to 16) and engaged in an average of three challenging behaviours across all live coaching sessions (range, 5 to 25). Across all five live coaching sessions, Daniel engaged in an average of 11 appropriate mands trained through FCT (range, 14 to 8) and exhibited an average of two challenging behaviours (range, 0 to 5). Catherina independently manded for a break on an average of 10 times over all live coaching sessions (range, 5 to 14) and engaged in zero challenging behaviours.

### Interventionist Treatment Fidelity

Figure 2 also displays the results of interventionist treatment fidelity during FCT delivered through live coaching condition. During baseline, all three interventionists completed 0% of the treatment fidelity checklist steps correctly. After the conclusion of didactic training and the completion of the first three coaching sessions, Fadimah and Majidah’s data were stable before illustrating an increasing trend. Due to school closures triggered by COVID-19, sessions ended abruptly for Jana after session three of FCT and further data were not collected; therefore, no clear trend in data is visible. During the following FCT sessions, both Majidah and Fadimah reached mastery (80% treatment fidelity over three consecutive sessions), with Majidah’s score remaining stable at 100% and Fadimah’s score improved substantially to 100% for three consecutive days.

## Discussion

The first aim of this research was to assess if interventionists could run a brief FA while coached by a BCBA^®^ via telehealth. Secondly, the study aimed to evaluate if telehealth can be an effective platform to conduct a brief FA of challenging behaviour, identifying the variables maintaining challenging behaviours. Interventionists were able to provide clear and concise information to the BCBA^®^ surrounding the variables maintaining challenging behaviour when a FAST was completed via video conferencing. Training interventionists in the procedures to implement a brief FA was conducted by the first author through didactic training that included both asynchronous online training and live coaching sessions that built upon mastered content and allowed the BCBA^®^ to model skills and answer interventionists’ questions. Interventionists successfully conducted a FA with the support of the first and second authors through live coaching. Results illustrated clear maintaining variables of challenging behaviours despite the duration of FAs being only between 45–90 min (45 min with Sasmita, 55 min with Catherina and 90 min with Daniel).

The results of our study are comparable to previous research investigating brief FA. For example, the timeframe in which the FAs were conducted is comparable to the literature surrounding brief FA being conducted in-person (Derby et al., 1997; Northup et al., 1991). Our results also extend findings from a previous study by showing that the function of challenging behaviour can be identified via a brief FA conducted by professionals, rather than by parents (Gerow et al., 2021a, b). Additionally the study extends the results of current literature suggesting that telehealth is an effective way to conduct a FA of challenging behaviour both in home and in school settings (Machalicek et al., 2009, 2010; Suess et al., 2016; Wacker et al., 2013). Future research should consider evaluating training interventionists in new functional assessment methodologies via telehealth such as the interview-informed synthesised contingency analysis and the practical functional assessment (Hanley et al., 2014) as opposed to traditional methodologies that might require more time. Such methodologies described by Hanley et al. (2014) would be particularly suited to those who engage in severe challenging behaviours such as self-injury and aggression and researching the effectiveness of these procedures via telehealth would be a welcomed addition to the literature.

The final aims of the research was to assess if interventionists could be trained to fidelity to implement FCT via telehealth while evaluating child outcomes on challenging behaviour. Data showed a clear functional relation, in that FCT was successful at decreasing challenging behaviours to zero and allowing two of the three interventionists to reach mastery in treatment fidelity. A substantial amount of published studies assessing telehealth have so far focused on parent training; therefore, it was promising to obtain equally positive results when training professionals working with individuals with ASD, adding to the current evidence on training via telehealth (Barretto et al., 2006; Gibson et al., 2010; Machalicek et al., 2010; Schieltz & Wacker, 2020). Both Sasmita and Catherina’s challenging behaviour decreased to zero and remained at this level until sessions abruptly ended due to the COVID-19 pandemic. Daniel also showed a descending trend. These substantial decreases in participants’ challenging behaviour from baseline to synchronous coaching suggest that further sessions would have further confirmed gains. Such positive results are clearly useful for clinicians aiming to transition their services from in-situ to online.

While our study has demonstrated effective results, it is not without its limitations. Firstly, as a result of school closures across the globe due to the COVID-19 pandemic we were unable to collect follow-up data to assess maintenance of newly acquired interventionist and child behaviours. Maintenance data are key in determining the long-term effects of interventions and future research should carefully assess maintenance of appropriate mands taught through FCT delivered via telehealth as well as maintenance of reductions in challenging behaviour. The study focused on training professionals working with children with ASD. While it is important that professionals receive adequate training in behaviour analysis, had this research focused on parent training, sessions more than likely would have still gone ahead in the home environment amidst school and clinic closures. Therefore, intervention would not have come to an end for the children and parents could continue implementing effective interventions to decrease challenging behaviour. This highlights the importance of training primary caregivers on their own or alongside professionals in order to maintain and generalise gains but also as a means to guarantee the continuation of intervention during holidays, sickness or other exceptional circumstances such as the current pandemic.

Secondly, although interventionists and children were of the same culture with the exception of Majidah and Daniel, the authors of the study were not part of the same culture of the participants. One potential limitation to the study was that the authors did not explicitly ask if any cultural adaptations to procedures should be made. When conducting research in an international setting, it is imperative for clinicians and researchers to ensure they are designing culturally adapted interventions. Additionally, the authors were not native speakers of the participants’ primary language. As a result, there may have been some language misunderstandings. For example, a spike in challenging behaviour was visible with Sasmita after the third session of synchronous coaching. Interventionists were instructed to give free access to preferred items when the child was on break. During this footage, we observed that preferred items were being held back from Sasmita which appeared to be a direct antecedent to challenging behaviour. After discussing this with Sasmita’s interventionist, Fadimah, it was clear that a misunderstanding was the reason why preferred items had been held back. As this was clarified during the next live coaching session, Sasmita’s challenging behaviour decreased to zero and remained this way for the rest of the coaching sessions. Future researchers need to be aware of language barriers and communication difficulties when working with interventionists and families from different cultures and may consider employing an interpreter to facilitate understanding (Tsami et al., 2019). However, as consecutive interpretation might make synchronous coaching more time consuming and introduce confounding barriers when the interpreter is not a behaviour analyst, research and clinical teams should ideally seek to involve native speakers of the language employed by interventionists and families, whenever this is possible, a procedure that would also enhance culturally sensitive interventions.

When examining treatment fidelity, interventionists successfully met the mastery criterion of 80% or above in the short number of sessions conducted, suggesting that disseminating interventions to an international level can be done successfully. Jana, however, although she met the 80% criterion initially (session one), her score decreased from 100 to 66% after the completion of the third live coaching session. This was due to her not immediately reinforcing Catherina’s mands but rather presenting tasks and saying “First complete the task, then you can have your break”. Although fading reinforcement is an important element of any FCT intervention, thinning reinforcement on the third session was not part of our treatment fidelity checklist, but rather, interventionists were required to reinforce the appropriate mand on a fixed ratio of one. If coaching had continued with Jana, support provided during live sessions would have enabled her to reinforce newly acquired mands with fidelity. Future research should evaluate how interventionists can be trained on how and when to thin the reinforcement schedule (i.e., once the initial mastery criterion has been met; Wacker et al., 2013). Fadimah, although she reached the mastery criterion, her score fell by 20% on the final two sessions (still within mastery criterion). This was due to her presenting a few demands during the break contingency. For example, Fadimah would present Sasmita with demands such as “give me high five” or “what do you want to play?”. These demands did not act as an antecedent to challenging behaviour, as Sasmita’s behaviours remained at zero, but should have been avoided during the break, when interventionists are expected to follow the child’s lead. Further training on appropriate contingencies to present during the reinforcement period should be considered. Additionally, for child target behaviours we recommend a higher mastery criterion of 90% or above for future research and clinical practice, as this would facilitate skill maintenance and generalisation. Finally, our study did not collect data on the treatment fidelity of the BCBA^®^’s behaviours when coaching interventionists through the FA, nor on interventionists implementation as they were being coached. It is essential that in future studies treatment fidelity is measured across all phases of the study including during brief FAs.

The study has proven that telehealth certainly has its advantages, particularly for training professionals in international contexts in behaviour analysis. However, as recognised by Lerman et al. (2020), technology often does not come without its disadvantages. A barrier that became evident was the angle the camera was placed at. Quite often, the child would run out of shot, leading to difficulties in data collection. An initial ‘set-up’ session is recommended before data collection starts to ensure proper positioning of the camera and testing of internet connection and sound. On three occasions, sessions were interrupted due to a low connection speed. Switching to a mobile data connection was the solution for this. Future researchers should ensure participants have an internet speed of at least 5–8 megabits per second (Mbps) for high-definition video-conferencing streaming and consultants should ensure they have a download speed of 50Mbps or above for downloading large video files. Future researchers also need to be aware of other barriers surrounding a telehealth-based intervention, one of which includes additional time needed to prepare equipment. Interventionists within this study all had a strong technical knowledge and were able to set up and navigate equipment easily. However, clinicians and researchers should note that not all interventionists may have this knowledge and might therefore need additional support before starting the intervention. For example, researchers should be prepared to send a detailed task analysis on how to use the equipment (Lee et al., 2015; Lerman et al., 2020).

The current study aimed to train professionals working with children with ASD in assessment and intervention procedures aimed at decreasing challenging behaviour via telehealth on an international context. The study was successful in its aims with collected data showing clear maintaining variables of challenging behaviour through conducting a brief FA via telehealth, adding to the existing literature. Therefore, it is possible to conduct brief FAs via telehealth when a BCBA^®^/BCBA-D^®^ with extensive experience in FA is present and interventionists have received high quality training on FA. Secondly, two out of three interventionists met treatment fidelity when implementing FCT and all three children showed reduced or zero levels of challenging behaviour and an increase in mands. Sessions came to an end earlier than planned due to school closures as a result of the COVID-19 pandemic, however, the study has shown that telehealth can be effective and efficient when working with individuals globally who have difficulty accessing services due to the limited number of BCBAs^®^ in their countries. Overall, the results of our study have shown that training professionals working with individuals with ASD via telehealth when distance may be a barrier to accessing services is effective and feasible.

## Data Availability

Raw data are available upon request from the corresponding author.
